# Psychological impact of polygamous marriage on women and children: a systematic review and meta-analysis

**DOI:** 10.1186/s12884-021-04301-7

**Published:** 2021-12-13

**Authors:** Ismail Shaiful Bahari, Mohd Noor Norhayati, Nik Hussain Nik Hazlina, Che Abd Aziz Mohamad Shahirul Aiman, Nik Ahmad Nik Muhammad Arif

**Affiliations:** 1 Department of Family Medicine, School of Medical Sciences, Universiti Sains Malaysia, Health Campus, 16150 Kubang Kerian, Kelantan Malaysia; 2 Women’s Health Development Unit, School of Medical Sciences, Universiti Sains Malaysia, Health Campus, Kubang Kerian, Kelantan Malaysia

**Keywords:** Polygamy, Impact, Psychological, Social, Women, Children

## Abstract

**Background:**

Over the last two decades, there has been significant growth in public, political, and academic awareness of polygamy. Polygamous families have distinct household problems, usually stemming from jealousy between co-wives over the husband’s affections and resources. This study aimed to ascertain the psychological impact of polygamous marriage on women and children worldwide.

**Methods:**

A systematic search was performed in MEDLINE (PubMed), Scopus, CINAHL (EBSCOhost), Google Scholar, and ProQuest using search terms such as “marriage” and “polygamy.” Studies published from the inception of the respective databases until April 2021 were retrieved to assess their eligibility for inclusion in this study. The Joanna Briggs Institute Critical Appraisal Checklist was used for data extraction and the quality assessment of the included studies. The generic inverse variance and odds ratios with 95% confidence intervals (CI) were calculated using RevMan software.

**Results:**

There were 24 studies fulfilling the eligibility criteria, and 23 studies had a low risk of bias. The pooled meta-analysis showed women in polygamous marriages had a 2.25 (95% CI: 1.20, 4.20) higher chance of experiencing depression than in monogamous marriages. Children with polygamous parents had a significantly higher Global Severity Index with a mean difference of 0.21 (95% CI: 0.10, 0.33) than those with monogamous parents.

**Conclusions:**

The psychological impact of polygamous marriage on women and children was found to be relatively higher than monogamous marriage. Awareness of the proper practices for polygamy should be strengthened so that its adverse effects can be minimized. The agencies involved in polygamous practices should broaden and enhance their understanding of the correct practice of polygamy.

**Supplementary Information:**

The online version contains supplementary material available at 10.1186/s12884-021-04301-7.

## Background

Polygamy may create a complex family system involving the husband’s relationship and relations between subsequent wives and children [1]. Polygamous families have distinct household problems, usually stemming from jealousy between co-wives over the husband’s affections and resources [2]. In addition to studies documenting polygamy’s detrimental effects on wives’ health, researchers have identified polygamy as a risk factor for adverse child health outcomes [3].

Polygamy is defined as “a marital relationship involving multiple spouses” [4]. There are three types of polygamy: polygyny refers to “one husband [who] is married to two or more wives,” polyandry refers to “one wife married to two or more husbands,” and polygynandry refers to “a group marriage scenario in which two or more wives are simultaneously married to two or more husbands” [4]. Only 2% of the global population practices polygamy. Polygamy is most often found in West and Central Africa, which the highest was in Burkina Faso (36%) with widespread among people who practice folk religions (45%), Muslims (40%), and Christians (24%) [5].

A recent systematic review had confirmed that children from polygamous marriages experienced physical and emotional abuse associated with parental neglect and abuse [6]. A qualitative study on female children and young adults found that polygamous marriage formed an emotional abuse to the daughters since they have witnessed the mother’s severe pain of second marriage and ascribe the mother’s pain to it [7]. These abuses may be associated with more mental health problems, social problems, and lower academic achievement in children from polygamous marriages compared to monogamous marriages [8].

In a qualitative study of American Muslims of various ethnic backgrounds, women in polygamous relationships have reported being abused by their husbands or other wives [3]. The prevalence of emotional distress (86.8%), fearful feeling (17%), low self-esteem (58.4%), and loneliness (64.1%) have also been found higher among women in polygamous relationships compared to monogamous marriages with the prevalence of 17.9, 7.7, 7.7, and 12.8%, respectively in Bedouin-Arabs of the Negev region in Israel [9] In polygamous marriages, where the mother is the first wife, the environment at home is stressful, parental investment is low, and resources are diluted; however, studies on polygamy and associated fertility issues have been mixed [10].

Polygamous women are genuinely at risk of experiencing psychological and emotional distress. For example, one study found that women in polygamous marriages are at a higher risk of low self-esteem and depression than women in monogamous relationships and enjoy less marital satisfaction and more problematic mother-child relationships [11]. There were significant differences between women in polygamous and monogamous marriages. There was a higher prevalence of somatization, depression, anxiety, hostility, paranoid ideation, psychoticism, general symptom severity, positive symptoms total, and psychiatric disorder, as well as lower ratings of life and marital satisfaction, family functioning, and self-esteem among polygamous wives [12]. A recent study also demonstrated similar findings but showed no significant difference in women’s marital satisfaction between polygamous and monogamous marriages [13].

On the bright side, polygamy also demonstrated positive impacts. Childless wives are willing to have legal and valid polygamous marriages than the other wives to obtain offspring and descendants for the husband. Besides that, warmth and affection for polygamous families may provide positive role models for children’s mental health and self-esteem [14].

Determining the impact of polygamous marriage on women and children worldwide can provide a better assessment than discrete primary studies. Identifying this impact can help give a clear understanding and serve as the basis for the development of appropriate strategies that address primary prevention to counter the potential negative impact affecting women and children. This systematic review and meta-analysis aimed to ascertain the psychological impact of polygamous marriage on women and children worldwide. We have included both women and children because the impact of polygamous marriage might affect both groups. This review summarizes the available evidence, effect estimates, and strength of the statistical associations between polygamous and monogamous marriages and the psychological impact on women, and children.

## Methods

### Study design and search strategy

A systematic review and meta-analysis were conducted to assess the impact of polygamous marriages on women and children. The Preferred Reporting Items for Systematic Reviews and Meta-Analyses (PRISMA) guidelines were followed [15]. This review was registered in the PROSPERO database (CRD42021226530). The review followed the process outlined in the protocol. A systematic search for relevant articles was performed in the MEDLINE (PubMed), Scopus, CINAHL (EBSCOhost), Google Scholar, and ProQuest databases. The search was undertaken using descriptors such as “marriage” (MeSH terms) OR “polygamy” (text word) AND “women” (MeSH terms) AND “children” (MeSH terms). The search terms were flexible and tailored to the various electronic databases. Studies published from the inception of the respective databases until April 2021 were retrieved to assess their eligibility for this study. The reference lists of the included citations were cross-checked to find additional potentially eligible studies.

### Eligibility criteria

The inclusion criteria included studies that reported the psychological impact of polygamous marriage on women and children of all ages up to 18 years old. The Oxford dictionary defines psychological impact as involving the mental and emotional state of a person [16]. In this study, polygamy referred to “a marital relationship involving multiple wives” [4].

Studies with cross-sectional, case-control, and cohort designs published in English were included. Case series/reports, conference papers and proceedings, articles available only in abstract form, editorial reviews, letters of communication, commentaries, systemic reviews, and qualitative studies were excluded.

### Study selection and screening

**Fig. 1:**
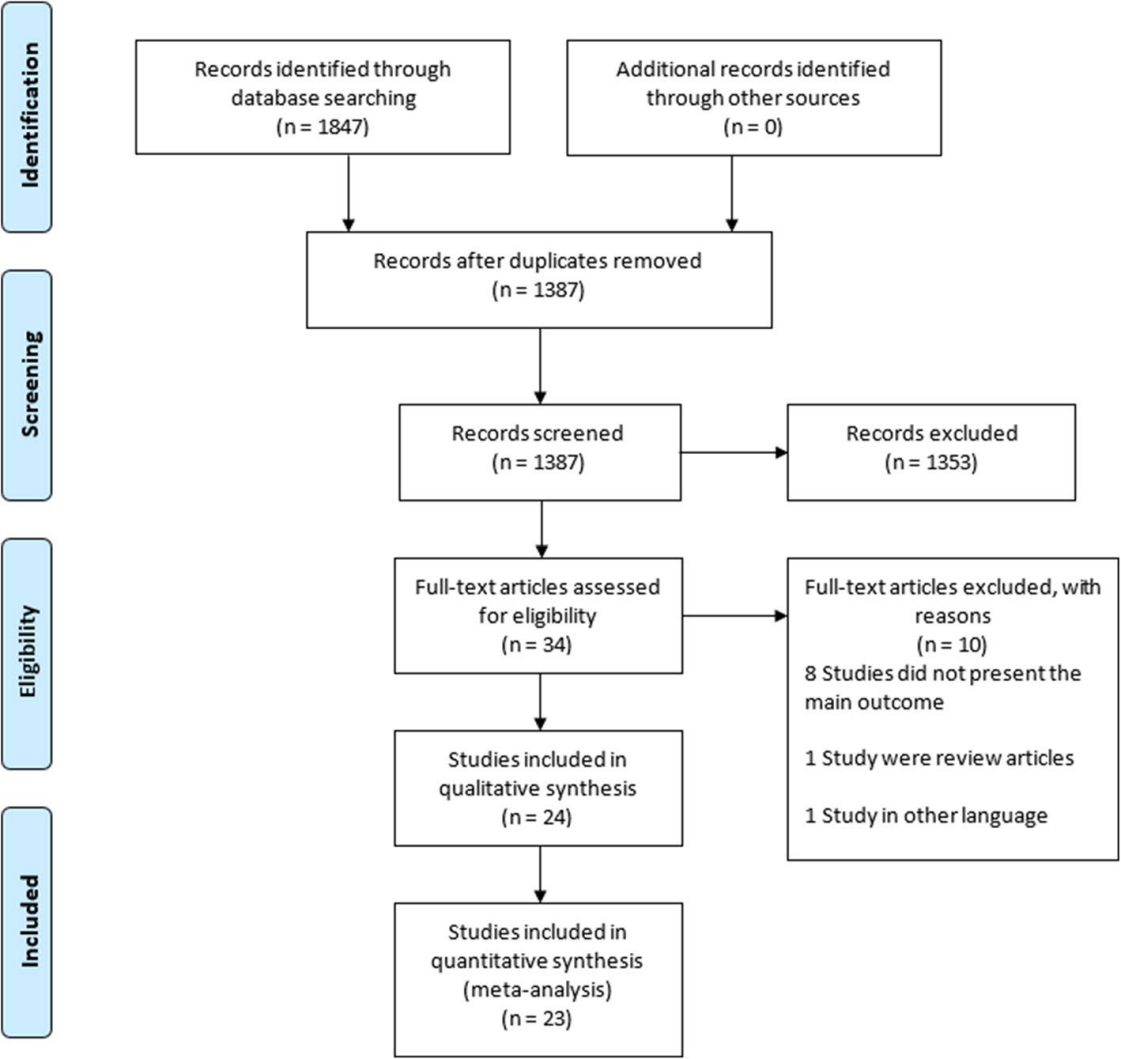
Prisma flow chart impact of polygamous marriage on women and children

All the records identified using our search strategy were exported to EndNote X8 software (Clarivate Analytics, Philadelphia, PA). Duplicate articles were removed. Two independent reviewers screened the titles and abstracts of the identified articles. The full texts of the eligible studies were obtained and read thoroughly to assess their suitability. A consensus discussion was held in a conflict between the two reviewers, and a third reviewer was consulted. The search method presented in the PRISMA flowchart **(**Fig. 1**)** shows the included and excluded studies, with reasons for the exclusions.

### Quality assessment and bias

Critical appraisal was performed to assess the data quality using the Joanna Briggs Institute Critical Appraisal Checklist for cross-sectional, case-control, and cohort studies [6]. Two reviewers performed the bias assessments independently. The risk of bias was considered low when more than 70% of the answers were “yes,” moderate when 50–69% of the answers were “yes,” and high when up to 0–49% of the answers were “yes.” Studies that showed a high or moderate risk of bias were excluded from the meta-analysis [17].

### Data extraction process

Two reviewers independently extracted data into Microsoft Excel 365 (Microsoft, Redmond, Washington). The process included the first author, publication year, study location, study design and setting, study population, sample size, impact, polygamy definition, and data for calculation of effect estimates for psychological impact. In the event of missing data, the authors were contacted to obtain further information.

### Results synthesis and statistical analysis

The prevalence outcomes of the total sample over the total population were reported as percentages, and the cumulative estimates were reported as odds ratios (OR) and mean differences (MD) with 95% confidence intervals (CI). The analysis was performed using RevMan software version 5.4 (Nordic Cochrane Centre, Copenhagen, Denmark). We used a generic inverse variance with a random-effects model to pool the data. The I^2^ statistic was used to assess heterogeneity. As a guide, I^2^ was interpreted as follows: 0–40% might not be important, 30–60% may represent moderate heterogeneity, 50–90% may represent substantial heterogeneity, and 75–100% indicated considerable heterogeneity [18]. The subgroup analyses were performed based on geographical regions if there was an adequate number of articles for each subgroup. Sensitivity analysis was conducted for studies with a wide range of confident intervals.

## Results

### Characteristics of the included studies

A total of 1847 articles were retrieved through the electronic database search using different search terms (Supplementary file 1), and 545 duplicated records were removed. The remaining 1387 articles were screened for eligibility. Among them, 1353 articles were excluded based on their titles and/or abstract evaluations. The full texts of 35 articles were searched. Subsequently, ten articles were excluded; where eight studies [19–26] did not present the main outcome, one study [14] was a review article, and one study [27] was in another language. Twenty-four studies underwent a quality assessment using Joanna Briggs Institute Critical Appraisal Checklist (Fig. 1, Supplementary file 2). Based on the quality assessment, 23 studies had a low risk of bias and one study had a moderate risk of bias [28]. All the low risk studies were cross-sectional and proceeded with quantitative assessment.

Among the 23 studies, 17 are about women [4, 9, 29–43], while six other studies focus on the children [44–49]. Among the studies, 11 of them is from Israel [4, 9, 30–32, 34, 44–46, 48, 49], three studies from Turkey [40, 41, 43], two studies from Iran [36, 38], a study from Uganda [29], a study from Nigeria [47], a study from Egypt [33], a study from UAE [37], a study from Syria [39], a study from Tanzania [42] and a study from Jordan [35]. The smallest sample size was 66 [38], and the largest was 2000 [35]. This study included 5963 women (Table 1) and 1567 children (Table 2).

**Table 1:** Summary of research articles (*n* = 18) on the impact of polygamous marriages on women

Authors	Study Area	Study design	Sample size (n)	Polygamous marriage (n)	Monogamous marriage (n)	Quality assessment (%)
Abbo 2008 [29]	Uganda	Cross-sectional	209	37	90	100
Al- Sherbiny 2005 [33]	Egypt	Cross-sectional	100	50	50	100
Daradkeh 2006 [35]	Jordan	Cross-sectional	2000	544	947	100
Hamdan 2008 [37]	United Arab Emirates	Cross-sectional	224	28	155	100
Kianpoor 2006 [38]	Iran	Cross-sectional	66	31	26	75
Maziak 2002 [39]	Syria	Cross-sectional	412	26	331	100
Ozkan 2006 [41]	Turkey	Cross-sectional	138	88	50	100
Patil 2008 [42]	Tanzania	Cross-sectional	408	96	312	87.5
Ozer 2013 [40]	Turkey	Cross-sectional	172	99	73	87.5
Farahmand 2019 [36]	Iran	Cross-sectional	398	248	150	100
Yilmaz 2018 [43]	Turkey	Cross-sectional	108	72	36	100
Daoud 2014 [34]	Israel	Cross-sectional	461	100	361	87.5
Al-Krenawi 2001 [4]	Israel	Cross-sectional	92	53	39	100
Al-Krenawi 2006 [9]	Israel	Cross-sectional	352	117	235	87.5
Al-Krenawi 2008 [32]	Israel	Cross-sectional	315	156	159	100
Al-Krenawi 2011 [31]	Israel	Cross-sectional	199	93	106	100
Al-Krenawi 2012 [30]	Israel	Cross-sectional	309	187	122	100
Chaleby 1985 [28]	Kuwait	Cross-sectional	125	31		62.5

**Table 2:** Summary of research articles (*n* = 6) on the impact of polygamous marriages on children

Authors	Study Area	Study design	Sample size (n)	Polygamous marriage (n)	Monogamous marriage (n)	Quality assessment (%)
Al-Krenawi, 2002 [44]	Israel	Cross-sectional	101	19	82	87.5
Al-Krenawi 2000 [45]	Israel	Cross-sectional	292	146	146	100
Al-Krenawi 2008 [46]	Israel	Cross-sectional	352	178	174	100
Bamgbade 2014 [47]	Nigeria	Cross-sectional	206	50	156	100
Hamdan, 2009 [49]	Israel	Cross-sectional	406	208	198	87.5
Elbedour 2003 [48]	Israel	Cross-sectional	210	114	84	75

### Prevalence of polygamy

**Fig. 2:**
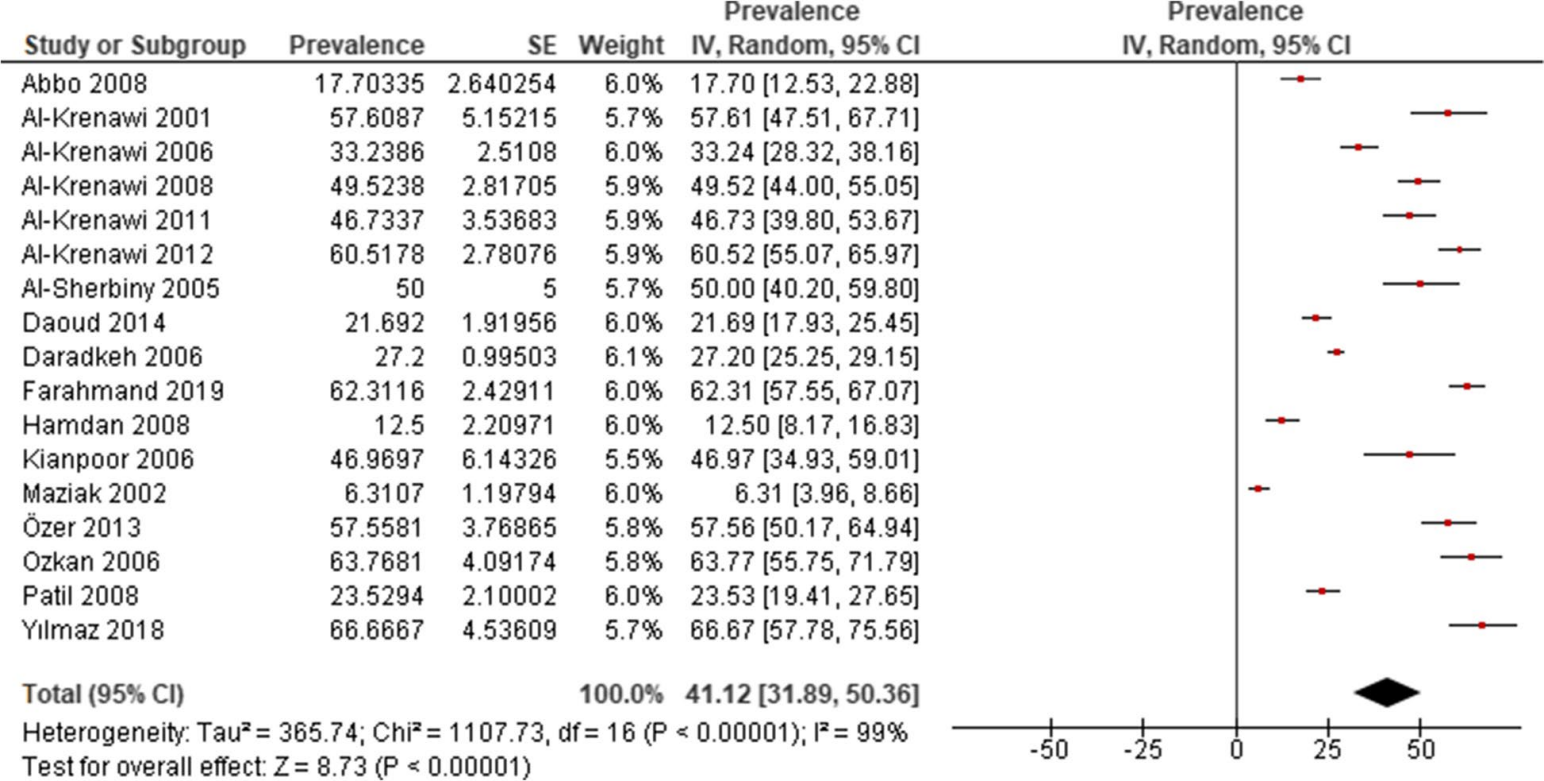
Prevalence of polygamy

Seventeen studies were included for estimation of the prevalence of polygamy in the women population [4, 9, 29–43]. A wide range was observed, ranging from 6.3% [39] to 66.7% [43]. The pooled prevalence of polygamy reported between 2001 and 2019, mainly in the middle-east region, was 41.12% (95% CI: 31.89, 50.36) (Fig. 2).

### Impact of polygamy on women compared to monogamy

**Fig. 3:**
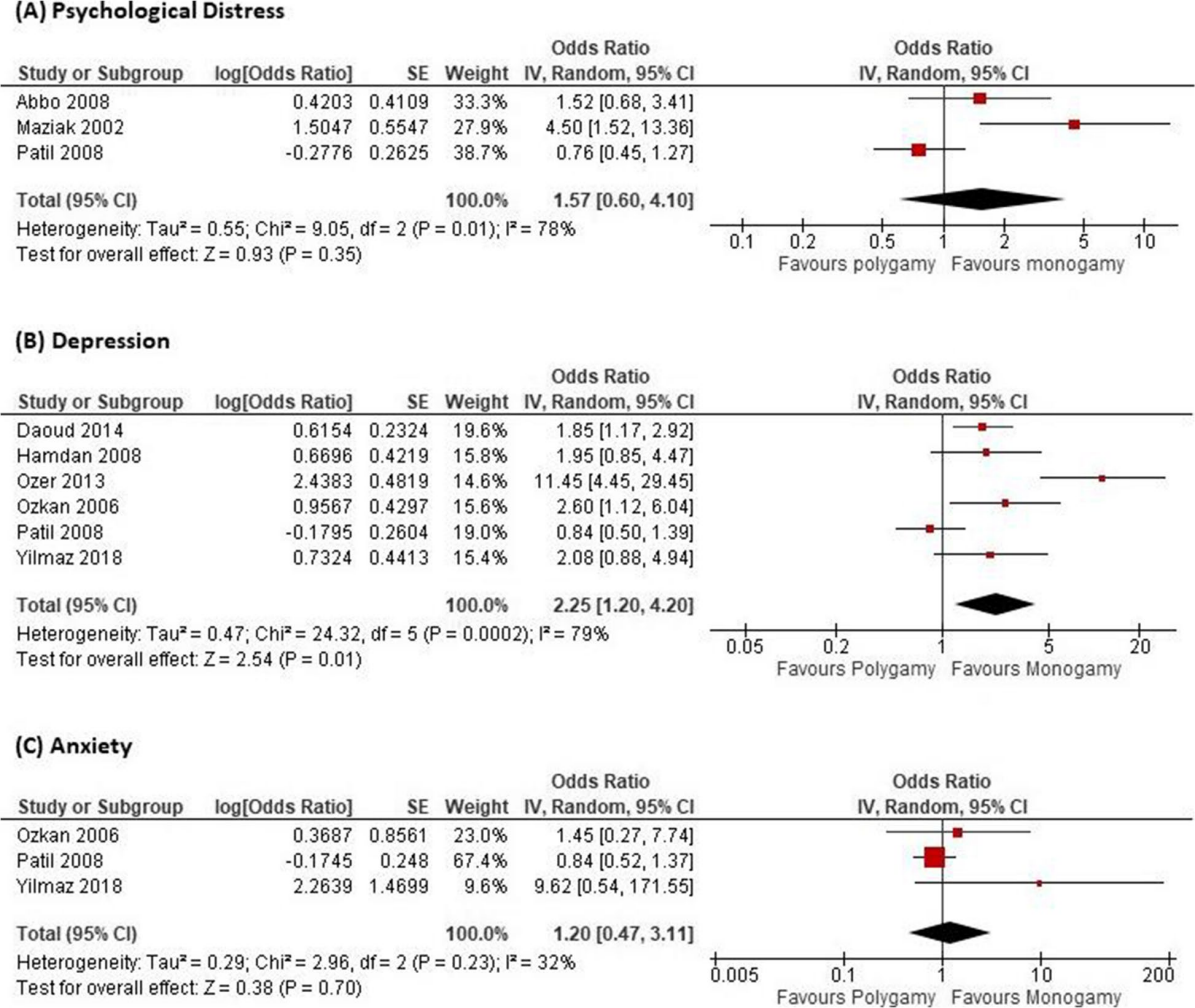
Forest plots for **A** psychological distress, **B** depression, and **C** anxiety among women in polygamous versus monogamous marriages

In this review, the psychological impact, including depression and anxiety, on women in polygamous marriages compared to monogamous marriages was evaluated. Only the pooled meta-analysis analysis for depression [34, 37, 40–43] showed a significant difference among women where it is 2.25 (95% CI: 1.20, 4.20) higher chance of experiencing depression in polygamous marriages compared to monogamous marriages. However, for psychological distress (OR 1.57 [95% CI: 0.60, 4.10]) [29, 39, 42] and anxiety (OR 1.20 [95% CI: 0.47,3.11]) [41–43] there were no significant difference between women in polygamous and monogamous marriages **(**Fig. 3**)**. Panic disorder, too, did not show a significant difference (OR 4.05 [95% CI: 0.71, 23.13]). Sensitivity analysis was conducted in the anxiety data due to the wide range of confident intervals in Yilmaz [43]. The estimated OR changed to 0.88 (95% CI: 0.55, 1.40) with I^2^ of 0%.

Four studies [9, 30–32] evaluated a broad range of psychological impact using the Symptom Checklist-90-Revised (SCL-90) instrument **(**Table 3**)**. The scores for somatization (MD 0.50 [95% CI: 0.28, 0.72]), obsessive-compulsive (MD 0.37 [95% CI: 0.09, 0.64]), interpersonal sensitivity (MD 0.41 [95% CI: 0.14, 0.67]), depression (MD 0.46 [95% CI: 0.16, 0.77]), anxiety (MD 0.49 [95% CI: 0.23, 0.75]), hostility (MD 0.49 [95% CI: 0.25, 0.73]), phobia (MD 0.39 [95% CI: 0.11, 0.67]), paranoia (MD 0.36 [95% CI: 0.20, 0.51]), and psychoticism (MD 0.42 [95% CI: 0.20, 0.64]) had significantly higher occurence in the women from polygamous marriages than monogamous marriages. Global Severity Index (GSI) for psychological dimensions is also higher in polygamous marriage compared to monogamous with a mean difference of 0.44 (95% CI: 0.20, 0.68). Furthermore, four studies [9, 30–32] also reported on family function by using McMaster Family Assessment Device (FAD) among women where polygamous marriage had shown a mean difference of 0.34 (95% CI: 0.20, 0.49) compared to monogamous marriages.

**Table 3:** Mean differences in the Symptom Checklist-90-Revised scores among women in polygamous marriages versus those in monogamous marriages in four studies [9, 30–32]

No.	Symptoms assessed by the Symptom Checklist-90-Revised	Mean difference (95% CI)	Heterogeneity	*P*-value of the overall effect
1	Somatization	0.50 (0.28, 0.72)	76%	< 0.001
2	Obsessive–compulsive behavior	0.37 (0.09, 0.64)	87%	0.009
3	Interpersonal sensitivity	0.41 (0.14, 0.67)	86%	0.003
4	Depression	0.46 (0.16, 0.77)	92%	0.003
5	Anxiety	0.49 (0.23, 0.75)	84%	< 0.001
6	Hostility	0.49 (0.25, 0.73)	82%	< 0.001
7	Phobia	0.39 (0.11, 0.67)	86%	0.007
8	Paranoia	0.36 (0.20, 0.51)	54%	< 0.001
9	Psychoticism	0.42 (0.20, 0.64)	81%	< 0.001
10	Global Severity Index	0.44 (0.20, 0.68)	86%	< 0.001

### Impact of polygamy on children compared to monogamy marriages

There were two studies [44, 46] which reported the impact of polygamy in the children in terms of psychological impact using the SCL-90 instrument **(**Table 4**)**. All scores for the psychological impact reported a slightly higher risk in children with parents practicing polygamy compared to monogamy where somatization (MD 0.20 [95% CI: 0.07, 0.34]), obsessive-compulsive (MD 0.27 [95% CI: 0.012, 0.42]), interpersonal sensitivity (MD 0.30 [95% CI: 0.14, 0.46]), depression (MD 0.22 [95% CI: 0.08, 0.37]), anxiety (MD 0.07 [95% CI: − 0.06, 0.20]) with *p* > 0.05, hostility (MD 0.24 [95% CI: 0.09, 0.39]), phobia (MD 0.33 [95% CI: 0.18, 0.49]), paranoia (MD 0.16 [95% CI: 0.01, 0.31]), and psychoticism (MD 0.28 [95% CI: 0.12, 0.43]). The GSI for children with polygamous parents have higher mean difference which is 0.21 (95% CI: 0.10, 0.33) compared to monogamous parents. In terms of social problems [44, 46], children with polygamous parents have higher risk of family dysfunction with MD 0.33 (95% CI: − 0.11, 0.77) compared to monogamous marriage. For school achievement, two studies [45, 46] reported children with polygamous parents had lower scores compared to monogamous parents and a study [47] reported that children with polygamous parents had difficulties in understanding subjects such as Mathematics and English.

**Table 4:** Mean differences in the Symptom Checklist-90-Revised scores among children with polygamous parents compared to monogamous parents in two studies [44, 46]

No.	Symptoms assessed by the Symptom Checklist-90-Revised	Mean difference (95% CI)	Heterogeneity	P-value of the overall effect
1	Somatization	0.20 (0.07, 0.34)	0%	0.003
2	Obsessive–compulsive behavior	0.27 (0.12, 0.42)	0%	< 0.001
3	Interpersonal sensitivity	0.30 (0.14, 0.46)	1%	< 0.001
4	Depression	0.22 (0.08, 0.37)	0%	0.003
5	Anxiety	0.07 (−0.06, 0.20)	0%	0.300
6	Hostility	0.24 (0.09, 0.39)	0%	0.002
7	Phobia	0.33 (0.18, 0.49)	0%	< 0.001
8	Paranoia	0.16 (0.01, 0.31)	0%	0.030
9	Psychoticism	0.28 (0.12, 0.43)	0%	< 0.001
10	Global severity index	0.21 (0.10, 0.33)	0%	< 0.001

## Discussion

The review was conducted to determine the psychological impact of polygamous marriage among women and children. The pooled prevalence of polygamous marriage in women from 17 studies was 41% (95% CI: 32, 50). Among women, depression was found to be significantly different between polygamous and monogamous marriages. Women and children in polygamous marriages have higher scores in somatization, obsessive-compulsive, interpersonal sensitivity, anxiety, hostility, phobia, paranoia, psychoticism, and GSI compared to monogamous marriages.

Various research reported that first wives in polygamous marriages would have a higher risk of depression, anxiety, and negative attitude [25, 38, 50, 51]. These researches reported similar findings as this current meta-analysis, where women in polygamous marriages have two times higher risk of developing depression compared to monogamous marriages. Al-Sherbiny [41] reported the “first wife syndrome,” where the first wife reported difficulties faced psychological, physical, and social problems among women in a polygamous marriage. This syndrome goes through a course of reaction where the initial response from the first wife after being informed of her husband’s remarriage is in the form of a nervous breakdown, emotional upset, or outburst of anger. Negative attitudes towards the husband and hostility towards the new wife always exist. After a lapse of time and gradual adaptation, these women reported that negative physical, psychological and social attitudes would decrease [33].

Al Krenawi [25] also reported that the transition from sole wife to senior wife is traumatic, leading to the senior wife having a loss of self-esteem. The Bedouin-Arabs of Negev showed that 58.4% of the polygamous wives had low self-esteem. This circumstance encouraged them to withdraw from their social networks, contributing to feeling lonely (64.1%) among these polygamous wives.

Women in polygamous marriages scored significantly higher in all psychological dimensions in the SCL-90: somatization, interpersonal sensitivity, depression, anxiety, phobic, paranoia, psychoticism, and GSI, and these findings were similar to a review [13]. Al- Issa [52] indicated that somatization might be more prevalent in the non-western world than in the west. This may be due to the ethnicity of Arabs, where exhibiting somatization behaviour is one of the major ways to express emotional distress [4, 23, 52]. In this culture, the first wife is usually not consulted when her husband s to remarry, leading to fewer familial, social, and economic resources where it can be distressing [53]. This would lead to first wives in polygamous marriages having more anxiety, psychoticism, paranoia, and feeling of powerlessness than the second and third wives [22]. Apart from that, this meta-analysis also reports that family functioning scores have been worse in women with polygamous marriages than monogamous marriages. It may be due to the husband’s attention being divided between two families; thus, economic resources became more diluted. One study reported that family functioning and financial status depend on one another, strongly associated with mental disorders [54]. A worsened family’s economic situation could lead to poorer family functioning [32].

Children with polygamous parents experienced more psychological impact compared to monogamous parents; however, these findings were limited to only two studies. A review based on five papers concluded that children from polygamous families had higher levels of psychological impacts than those from monogamous families [8]. Elbedour [10] suggested that polygamy effects on children are more noticeable and disappear as they grow older. Children in polygamy marriages will have lower academic achievement [6, 24, 25]. Still, children’s academic achievement may be less affected due to a better understanding of stressful events and more successful managing emotions [48]. Children from kindergarten through Grade 6 reported a lower level of education achievement based on the examination results. They had difficulty adjusting to their schools, thus indicating that these social problems were impacted by their parents’ polygamous marriage that has affected their formal education system [45]. The children of these marriages will have a huge disadvantage in their education and increase school dropouts.

The SCL-90 instrument performed on the children in polygamous marriages showed higher psychological impact scores in all nine domains [44, 46]. However, there may be an additional cultural impact on some of the domains. Research revealed that Arab children exhibit higher levels of depression compared to the control samples in the United States [55]. It also implied interpersonal sensitivity, where its risk increased in conjunction with the presence of depression [56]. Despite having parents with polygamous or monogamous marriages, family functioning plays a much more prominent role in children’s self-esteem, peer relation, and mental health [44, 46]. Findings indicated the impact of polygamy itself, but a well-functioning family will not impair children’s social adjustment and mental health [57]. Economic status also plays a significant role in family functioning and children’s mental health [46, 58]. Unfortunately, the children perceived that their parents’ polygamous marriages had made their families’ economic and family functioning worse [44, 46]. This plays a major role in dealing with children’s emotional and financial pressure.

This meta-analysis has a few limitations. Most studies have a very different range of tests and scales that hinder making a reasonable conclusion. The random-effects model assumes the presence of heterogeneity in which each study has its study-specific effect. However, subgroup analysis to explore the differences to understand the observed effect was not possible due to limited studies. This study is limited by only including studies published in the English language. Most studies were conducted in the Middle East, specifically Arab societies, limiting the results and comparisons. We could not deduce whether the impact is solely due to polygamous marriages or the culture of societies. All the included studies were of cross-sectional design. Due to its nature, temporal causation cannot be established.

## Conclusions

The psychological impact of polygamous marriage on women and children was relatively higher than monogamous marriage. This study also concluded that polygamous marriage plays a major role in the development of children not only mentally but also socially. Family functioning also has a major role in determining the outcome of polygamous impact on the population. Awareness of the proper practices for polygamy should be strengthened so that its adverse effects can be minimized. The agencies involved in polygamous practices should broaden and enhance their understanding of the correct practice of polygamy. It is also necessary for healthcare professionals to have a better evaluation for women and children in this family practice to provide them with a better quality of life. Polygamy should be recognized as a particular risk factor for developing social problems in children; thus, with proper education to the families, more attention to the children’s emotional and social needs is required to avoid this situation. Future studies on polygamous marriage should emphasize more on children with broader sampling across various cultures. These studies should also use standardized measuring tools to ensure a better conclusion.

## Supplementary Information

**Additional file 1.**

**Additional file 2.**

## Data Availability

All data are available within the manuscript.
