# Supplementary material for: Psychological impact of polygamous marriage on women and children: a systematic review and meta-analysis
Source: BMC Pregnancy Childbirth. 2021 Dec 13;21:823. doi: 10.1186/s12884-021-04301-7 (PMC8667458; doi:10.1186/s12884-021-04301-7)
Supplement: Supplementary file 1 — Additional file 1. [file 12884_2021_4301_MOESM1_ESM.pdf]

Supplementary file 1. Search strategies

Database	Search	Number of studies
Ebscohost	psychological AND polygamy AND (women or female or woman or females) OR (children or adolescents or youth or child or teenager) AND marriage AND depress* AND anxiety	1,511
Pubmed	(polygamy[Text Word] OR polygyny[Text Word] OR polygynous[Text Word]) AND ((("Women"[Mesh]) OR ((women[Text Word] OR woman[Text Word] OR wife[Text Word] OR wives[Text Word]))) OR ((("Child"[Mesh]) OR "Adolescent"[Mesh])) OR ((minors[Text Word] OR kid[Text Word] OR child*[Text Word] OR adolescen*[Text Word] OR youth*[Text Word] OR teen*[Text Word] OR pubescen*[Text Word]))))) AND (("Psychological Phenomena"[Mesh]) OR (psychological*[Text Word] OR psychopathological*[Text Word]))	121
Scopus	psychological AND polygamy AND (women OR female OR woman OR females) OR (children OR adolescents OR youth OR child OR teenager) AND marriage AND depress* OR anxiety AND impact	155
ProQuest	Polygamy OR marriage AND women AND children<br>Polygamy AND women AND children<br>Psychological impact AND Polygamy AND women AND children	5730<br>152<br>85
