# Supplementary material for: Psychological impact of polygamous marriage on women and children: a systematic review and meta-analysis
Source: BMC Pregnancy Childbirth. 2021 Dec 13;21:823. doi: 10.1186/s12884-021-04301-7 (PMC8667458; doi:10.1186/s12884-021-04301-7)
Supplement: Supplementary file 2 — Additional file 2. [file 12884_2021_4301_MOESM2_ESM.pdf]

Supplementary file 2. Quality assessment based on Joanna Briggs Institute Critical Appraisal Checklist

	Criteria (Yes/No/Unclear/NA)	Elbedour 2003	Al-Krenawi 2000	Bamgbade 2014	Abbo 2008	Al-Sherbiny 2005	Chaleby 1985	Darakeh 2006	Hamdan 2008	Kianpoor 2006	Maziak 2002	Ozkan 2006	Patil 2008	Al-Krenawi 2001	Al-Krenawi 2006	Al-Krenawi 2008	Al-Krenawi 2011	Yilmaz 2018	Al-Krenawi 2002	Ozer 2013	Farahmand 2019	Daoud 2014	Al-Krenawi 2012	Hamdan 2009
1	Were the criteria for inclusion in the sample clearly defin	1	1	1	1	1	0	1	1	1	1	1	1	1	1	1	1	1	0	1	1	1	1	1
2	Were the study subjects and the setting described in detail?	1	1	1	1	1	0	1	1	1	1	1	1	1	1	1	1	1	1	1	1	1	1	1
3	Was the exposure measured in a valid and reliable way?	1	1	1	1	1	1	1	1	1	1	1	1	1	1	1	1	1	0	1	1	1	1	1
4	Were objective, standard criteria used for measurement of the condition?	1	1	1	1	1	1	1	1	0	1	1	0	1	1	1	1	1	1	1	1	1	1	1
5	Were confounding factors identified?	0	1	1	1	1	1	1	1	1	1	1	1	1	1	1	1	1	1	1	1	1	1	1
6	Were strategies to deal with confounding factors stated?	0	1	1	1	1	0	1	1	0	1	1	1	1	0	1	1	1	1	0	1	0	1	1
7	Were the outcomes measured in a valid and reliable way?	1	1	1	1	1	1	1	1	1	1	1	1	1	1	1	1	1	1	1	1	1	1	1
8	Was appropriate statistical analysis used?	1	1	1	1	1	1	1	1	1	1	1	1	1	1	1	1	1	1	1	1	1	1	1
	**Total quality score**	6	8	8	8	8	5	8	8	6	8	8	7	8	7	8	8	8	6	7	8	7	8	8
	**Percentage**	75.00	100.00	100.00	100.00	100.00	62.50	100.00	100.00	75.00	100.00	100.00	87.50	100.00	87.50	100.00	100.00	100.00	75.00	87.50	100.00	87.50	100.00	100.00
	Risk of Bias: Rater I	low	low	low	low	low	mod	low	low	low	low	low	low	low	low	low	low	low	low	low	low	low	low	low
	Risk of Bias: Rater II	low	low	low	low	low	mod	low	low	low	low	low	low	low	low	low	low	low	low	low	low	low	low	low
	**Overall appraisal**	good	good	good	good	good	poor	good	good	good	good	good	good	good	good	good	good	good	good	good	good	good	good	good

JBI Quality Assessment Tool for cohort studies	
Criteria (Yes/No/Unclear/NA)	Al-Krenawi 2006
Were the two groups similar and recruited from the same population?	1
Were the exposures measured similarly to assign people to both exposed and unexposed groups?	1
Was the exposure measured in a valid and reliable way?	1
Were confounding factors identified?	0
Were the study subjects and the setting described in detail?	1
Were strategies to deal with confounding factors stated?	0
Were the groups/participants free of the outcome at the start of the study (or at the moment of exposure)?	1
Were the outcomes measured in a valid and reliable way?	1
Was the follow up time reported and sufficient to be long enough for outcomes to occur?	0
Was follow up complete, and if not, were the reasons to loss to follow up described and explored?	1
Were strategies to address incomplete follow up utilized?	1
Total quality score	8
Percentage	72.73
Risk of Bias: Rater I	Low
Risk of Bias: Rater II	Low
Overall appraisal	Good
